# Novel *MTTP* Gene Mutation in a Case of Abetalipoproteinemia with Central Hypothyroidism

**DOI:** 10.4274/jcrpe.galenos.2019.2019.0144

**Published:** 2020-11-25

**Authors:** Pembe Soylu Ustkoyuncu, Songül Gokay, Esra Eren, Durmus Dogan, Gokce Yıldız, Aysegul Yılmaz, Fatma Turkan Mutlu

**Affiliations:** 1 Kayseri City Hospital, Clinic of Pediatric Nutrition and Metabolism, Kayseri, Turkey; 2 Kayseri City Hospital, Clinic of Pediatric Gastroenterology, Hepatology and Nutrition, Kayseri, Turkey; 3 Kayseri City Hospital, Clinic of Pediatric Endocrinology, Kayseri, Turkey; 4 Kayseri City Hospital, Clinic of Pediatrics, Kayseri, Turkey; 5 Kayseri City Hospital, Clinic of Pediatric Genetic, Kayseri, Turkey; 6 Kayseri City Hospital, Clinic of Pediatric Hematology and Oncology, Kayseri, Turkey

**Keywords:** Abetalipoproteinaemia, central hypothyroidism, MTTP gene, novel mutation

## Abstract

Abetalipoproteinaemia (ABL) is an autosomal recessive disorder characterized by very low plasma concentrations of total cholesterol and triglyceride (TG). It results from mutations in the gene encoding microsomal TG transfer protein (MTTP). A nine-month-old girl was admitted to hospital because of fever, cough, diarrhea and failure to thrive. She had low cholesterol and TG levels according to her age. The peripheral blood smear revealed acanthocytosis. Thyroid function test showed central hypothyroidism. Cranial magnetic resonance imaging revealed the retardation of myelination and pituitary gland height was 1.7 mm. A homozygous novel mutation [c.506A>T (p.D169V)] was detected in the *MTTP* gene. Vitamins A, D, E, and K and levothyroxine were started. The coexistence of ABL and central hypothyroidism has not previously been reported. A homozygous novel mutation [c.506A>T (p.D169V)] was detected in the *MTTP* gene.

**What is already known on this topic?**

The coexistence of abetalipoproteinaemia (ABL) and peripheral hypothyroidism has been previously reported.

**What this study adds?**

The coexistence of ABL and central hypothyroidism has not been reported previously. A homozygous novel mutation [c.506A>T (p.D169V)] was detected in the MTTP gene. Our patient had dysmorphic features which is very rare in cases of ABL.

## Introduction

Abetalipoproteinaemia (ABL) (ABL; OMIM 200100) is an autosomal recessive disorder characterized by very low plasma concentrations of total cholesterol (TC) and triglyceride (TG). The disorder was first described in 1950 by Bassen and Kornzweig (1) in a patient with atypical retinitis pigmentosa. ABL results from mutations in the gene encoding microsomal TG transfer protein (MTTP). Patients with ABL often present with a range of symptoms such as failure to thrive, steatorrhea, hepatomegaly, loss of night and/or color vision, acquired atypical pigmentation of the retina, spinocerebellar ataxia, coagulopathy and myopathy, including fat malabsorption and manifestations of fat soluble vitamin deficiencies (2,3). Early detection and a low fat diet with fat soluble vitamin supplementation can prevent the neurological and ophthalmological complications (4).

Here, we report the coexistence of ABL and central hypothyroidism. This has not been previously reported. In addition, this patient had dysmorphic features. Coexistence of ABL and dysmorphic features is very rare. We detected a novel homozygous mutation in the *MTTP* gene.

## Case Report

A nine-month old girl was admitted to our hospital because of fever, cough, diarrhea (11 or 12 episodes a day), and failure to thrive. She was born by normal delivery at term with a birth weight of 3200 grams after an uneventful pregnancy. The patient was the second child of a non-consanguineous Turkish couple, who also had a 5-year-old healthy daughter.

At presentation her body weight was 4.7 kg [standard deviation (SD): -3.5], height was 62.5 cm (SD: -1.7), head circumference was 39 cm (3% percentile). Relative index was 71.2.

**Figure 1:**
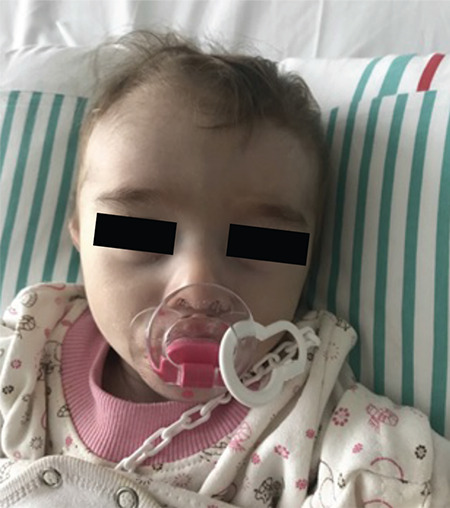
Dysmorphic features including hypertelorism, frontal bossing, triangular face and retromicrognaty

She was pale, her hair was thin and weak, her subcutaneous adipose tissue was decreased. She had rales in the middle zone of her left lung. Her abdomen was distended and bowel loops were prominent. She also had umbilical and bilateral inguinal hernia. She had dysmorphic features including hypertelorism, frontal bossing, triangular face and retromicrognathia (Figure 1).

Her head control was complete but she could not sit without support. Deep tendon reflexes were normoactive.

Laboratory investigations revealed: hemoglobin level was 9.8 g/dL; leukocyte count was 14820/mm^3^; and platelet count was 363000/mm^3^. Serum transaminases were mildly elevated with aspartate aminotransferase of 105 U/L, (normal range: 0-33) and alanine aminotransferase of 112 U/L (normal range: 0-32).

Vitamin D concentration was 11.6 mg/L (normal >30), alkaline phosphatase concentration was 99 u/L (normal range: 142-335), calcium concentration was 9.03 mg/dL (normal range: 8.6-10.2), phosphorus concentration was 3.15 mg/dL (normal range: 2.45-4.5) and parathyroid hormone concentration was 60 mg/L (normal range: 15-65).

**Figure 2:**
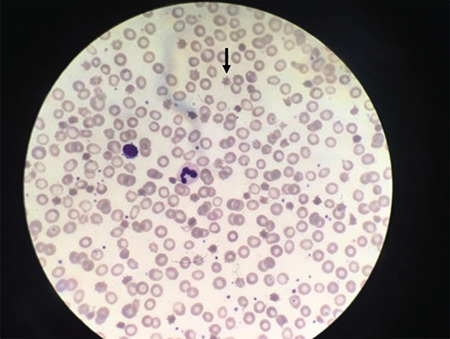
Acanthocytosis in the peripheral blood smear

TORCH screen was negative and other infections due to hepatotropic viruses or human immunodeficiency virus were ruled out. The results of coagulation tests, renal functions and electrolytes were also normal. She was hospitalized three times for bronchiolitis. Cystic fibrosis was considered in this patient due to malnutrition, recurrent bronchiolitis and elevation of liver function tests. Molecular genetic analysis of the *CFTR* gene was normal. Stool examination revealed no reducing substances and showed fat droplets. The search for pathogenic bacteria or parasites was negative. Normal levels of anti-endomysial antibodies ruled out celiac disease and basic metabolic tests including ammonia, lactate, pyruvate, blood acyl carnitine profile and amino acid analysis, urinary organic acid analysis, homocysteine and biotinidase activity were all normal. Congenital immune deficiency was ruled out. Immunoglobulin (Ig) profile (quantitative measurement of IgA, IgM, IgG and IgE), CD markers (CD3, CD19, CD56) and fagotest were normal. She had low cholesterol and TG concentrations with TC of 26 mg/dL (normal range: 3-200) and TG of 9 mg/dL (normal range: 0-200) according to her age. The peripheral blood smear revealed acanthocytosis (Figure 2).

The concentrations of vitamin E at 0.87 mg/L (normal range: 6.6-14.3), vitamin A at 71 ug/L (normal range: 316-820) and vitamin D at 11.6 ug/L (normal values >30) were very low. ABL was considered in this patient with these clinical and laboratory findings. Abdominal ultrasonography revealed multiple small stones (<3 mm) in both kidneys and ophthalmologic examination was normal.

Vitamin A (200 IU/kg/day), D (1200 IU/day), E (100 IU/kg/day), and K (5 mg/week) were started. High caloric (150 kcal/kg/day), low fat diet (15 %) with medium chain TG and Basic F^®^ formula were also started.

Thyroid stimulating hormone (TSH) was 1.4 mU/L (normal range: 0.73-8.35) and thyroxine (T4) level was 8.7 ng/L (normal range: 9.2-19.9). Control TSH level was 3 mU/L, and T4 level was 7.4 ng/L. Therefore, levothyroxine (12.5 mcg/day) was started. After the treatment, thyroid function tests were studied intermittently and levothyroxine dose was increased to 37.5 mcg/day. Free triiodothyronine (fT3) level was 3.55 ng/L (normal range: 2.15-5.83).

**Figure 3:**
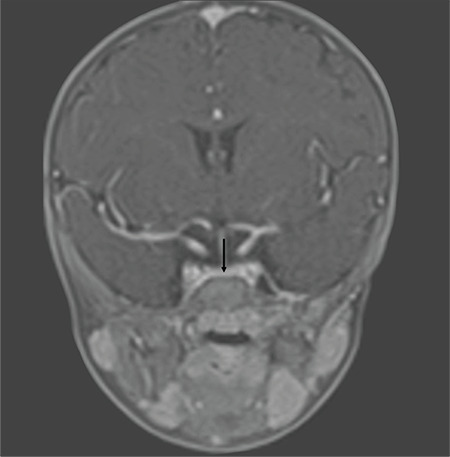
The appearance of pituitary gland

**Figure 4:**
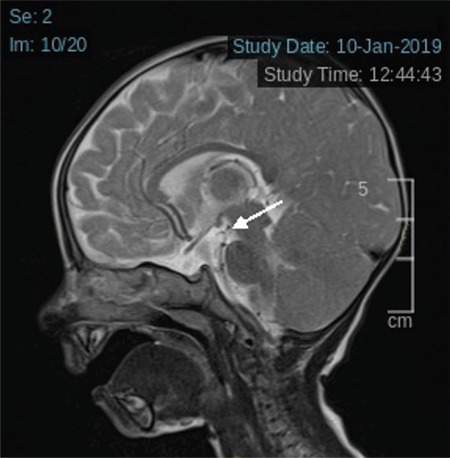
Sagittal magnetic resonance imaging image of the pituitary gland

Follicle-stimulating hormone, luteinizing hormone, adrenocorticotropic hormone (ACTH), cortisol and prolactin levels were evaluated for multiple pituitary insufficiency in addition to central hypothyroidism. Low dose ACTH stimulation test was performed due to a basal cortisol concentration of 10.1 mg/dL (normal >15). The peak cortisol values on low dose ACTH stimulation test were 35.8 mg/dL and evaluated as an adequate cortisol response. Prolactin concentration was 15.12 mg/L and accepted as normal. Insulin-like growth factor-1 (IGF-1) concentration was 8.53 ng/mL (SD -4.28). This low IGF-1 level was attributed to malnutrition. IGF-1 evaluation was planned according to anthropometric follow-up. Cranial magnetic resonance imaging (MRI) revealed the retardation of myelination and pituitary gland height was 1.7 mm (Figure 3). A further sagittal MRI view of the pituitary gland is shown in Figure 4.

Endoscopy was performed to support the diagnosis due to delay in obtaining the molecular genetic analysis. Macroscopic findings of endoscopy included normal mucosa of esophagus and stomach but “snow-like” appearance and pathologic findings were found in duodenum. Microscopic study showed widespread intracytoplasmic vacuolized degeneration of the villi.

*MTTP* gene analysis revealed a novel homozygous pathogenic variant [c.506A>T (p. D169V)]. *In silico* analysis indicated that the D169V substitution in *MTTP* was probably damaging. She is now 15 months old, her body weight was 8.9 kilograms (10-25% percentile) and height was 72.5 cm (3% percentile). She is receiving vitamin A (250 IU/kg/day), vitamin D (1200 IU/day), vitamin E (150 IU/kg/day), vitamin K (5 mg/week) and levothyroxine (37.5 mcg/day). She continues with a low-fat (15 %) and high-calorie diet. Vitamin A and E concentrations, together with thyroid function (TSH=3.9 mU/L, T4=14 ng/L) and coagulation tests, were normal. Stool number decreased significantly (2 or 3 episodes a day). Neurological examination is improved and she is standing and walking with support. This case report was written after receiving informed consent from the family.

## Discussion

Homozygous hypolipoproteinemia, and chylomicron retention disease have similar clinical findings with ABL. The diagnosis of ABL appeared to be the most likely in view of the normal plasma levels of TC and TG levels found in the parents, which suggested an autosomal recessive transmission. Our patient had also low levels of TG. For this reason, we sequenced the *MTTP* gene. *MTTP* gene analysis revealed a homozygous novel mutation [c.506A>T (p. D169V)].

More than 30 mutations in *MTTP* have been identified. The majority are point mutations resulting in either splicing errors or premature truncations (5). Our case had a point mutation in *MTTP*. 

There were some clinical features rarely associated with ABL in the present case. Facial dimorphism and psychomotor retardation have not often been described. It has been suggested that psychomotor retardation occurs due to hypothyroidism. Hasosah et al (6) reported dysmorphic features including hypertelorism, short nose, long philtrum, and thin upper lip in an 18-month-old male ABL patient.

Fat malabsorption causes a combination of unabsorbed fatty acids with calcium ions in the intestinal lumen leading to excessive absorption of oxalate. Rashtian et al (2) reported nephrolithiasis in a 12-months-old male infant, similar to the findings in our patient.

Hypothyroidism can be associated with ABL. Al-Mahdili et al (7) reported a mild case of ABL in association with subclinical hypothyroidism in a 32-year-old female. However, coexistence of ABL and central hypothyroidism has not been previously reported.

Euthyroid sick syndrome (ESS) is characterized by modification of thyroid hormone homeostasis due to non-thyroidal diseases. ESS has been described in liver disease, renal failure, after stress or surgery, in malnutrition or in malignancies. ESS is present if free fT3 was below the lower limit and free T4 was within the normal or low limits, while TSH was in the normal range (8). fT3 level of our patient was 3.55 ng/L (normal range: 2.15-5.83). Therefore, ESS was ruled out.

Krysiak and Okopie (9) reported that untreated or poorly managed ABL can impair the production of steroid hormones and cause some endocrine disorders such as chronic adrenal failure and hypergonadotropic hypogonadism.

Illingworth and Orwoll (10) reported that suboptimal response to corticotrophin stimulation maintained stable levels of plasma cortisol and showed no evidence of adrenal insufficiency with prolonged corticotrophin stimulation in ABL and hypobetalipoproteinaemia. The same group also showed that (11) a total absence of low density lipoprotein (LDL) does not impair adrenal steroidogenesis in the basal state and highlighted that plasma LDL serves as an important source of cholesterol for adrenal corticosteroid synthesis under conditions of sustained stimulation with ACTH (12).

Triantafillidis et al (13) and Illingworth et al (14) reported that patients with ABL have reduced levels of progesterone. This was attributed to low levels of serum LDL cholesterol. Reduced levels of leptin and IGF-1 are probably attributed to the impairment of nutritional status. Arem et al (15) reported that severe LDL cholesterol insufficiency impairs the initial glucocorticoid response to ACTH stimulation, but not overall cortisol production during sustained ACTH stimulation. Severe LDL cholesterol insufficiency may also contribute to the reduction of testosterone in chronically ill patients.

Ocular manifestations are variable, retinitis pigmentosa, ophthalmoplegia, ptosis, nystagmus, peripapillary chorioretinal degeneration, macular atrophy have been reported (16,17). The absence of ocular manifestations in our patient was attributed to the fact that they may appear at any time during the first two decades of life.

Hepatic involvement may include steatosis and elevated serum transaminase levels. In a few cases of ABL, hepatic injury progressed to fibrosis and cirrhosis, requiring transplantation (4,18). The hepatic manifestation in our patient was limited to elevated levels of serum transaminases.

## Conclusion

ABL is a rare disease of lipoprotein metabolism. Symptoms can be debilitating in most patients. Life expectancy is reduced without treatment. The coexistence of the disorder and central hypothyroidism has not been previously reported. ABL in this case was due to a novel homozygous mutation in the *MTTP* gene.
